# Translating PEARLS: Lessons Learned from Providers and Participants

**DOI:** 10.3389/fpubh.2014.00256

**Published:** 2015-04-27

**Authors:** Mark B. Snowden, Lesley E. Steinman, Pamela Piering, Sluggo Rigor, Andrea Yip

**Affiliations:** ^1^University of Washington Health Promotion Research Center, Seattle, WA, USA; ^2^Healthy Aging Consultant, Seattle, WA, USA; ^3^International Drop-In Center, Seattle, WA, USA; ^4^Seattle-King County Aging and Disability Services, Seattle, WA, USA

**Keywords:** evidence-based programs, RE-AIM, dissemination, implementation, depression, healthy aging, community-based

The Program to Encourage Active, Rewarding Lives (PEARLS) began 15 years ago when the director of our local area agency on aging (AAA) approached the University of Washington Health Promotion Research Center (HPRC). She was looking for a way to serve older adults with depression, including those served by the agency’s home and community-based services (HCBS) program. Depression in this population is high, when we analyzed data from 16,032 elders receiving HCBS in Washington State in 2005, two-thirds met criteria for clinical depression (1). This partnership between the university and local aging service providers created PEARLS, a brief, home-based program to teach people tools to effectively tackle the things in their lives that overwhelm them, and to in turn, improve their depressive symptoms. These tools include a seven-step approach to problem-solving and action planning to increase physical, social, and pleasant activities. PEARLS is a structured intervention delivered in 6 to 8 one-hour visits over the course of a 4- to 5-month period. Sessions are tapered from weekly to monthly to give participants an opportunity to practice and learn the skills. More information about the program can be found at www.pearlsprogram.org.

The Program to Encourage Active, Rewarding Lives (PEARLS) reduced depression and improved quality of life in two randomized controlled trials (2, 3). Since then, UW HPRC continues to work with the local AAA and other sites to help translate the evidence-based program into everyday practice. The implementation challenges are striking given that PEARLS includes several ingredients for program success: it was designed with an adopting organization as a key partner, the model trains existing staff to deliver PEARLS so new staff do not need to be hired, and the program is successfully funded in some locations through several diverse funding streams. We have learned a lot from organizations and staff that deliver PEARLS through our monthly technical assistance calls and other dissemination research and activities. We have also learned a lot from program participants through PEARLS sessions, focus groups and interviews. A selection of key learnings is provided below, organized by Glasgow’s RE-AIM framework to help improve the success of evidence-based program delivery in “real-world” settings (4–6). This framework consists of five elements – reach, effectiveness, adoption, implementation, and maintenance – that present the overall public health impact of a program or policy. It is important for programs to perform well across each of these five elements in order to maximize overall impact (7).

## Reach

Recruitment is an ongoing implementation challenge for PEARLS. Previous data suggest that 10% of eligible participants are referred to PEARLS and 50% of those are enrolled in the program (8, 9). Barriers exist for both those tasked with recruiting participants and for those invited to participate in the program. Successful PEARLS programs engage a range of community-based providers to refer to PEARLS that is similar to the gatekeeper approach used in other mental health programs (10). Many people that touch a potential participant’s life are appropriate referral sources – from the Meals on Wheels delivery person to the resident services coordinator in a low-income housing facility. Participants have shared that having a trusted person – whether a familiar case worker or pastor – make the referral is particularly important when discussing a sensitive subject such as depression (9). These gatekeepers can be trained to administer a brief validated depression screen such as the two-item patient health questionnaire (PHQ-2) (11).

In addition to provider engagement, it is essential to use culturally appropriate materials and media for the target community. Strategies include putting photos of PEARLS counselors on recruitment flyers and publishing participant stories in newsletters, community papers, or in digital form (12, 13). Former PEARLS participants agree that the “best way to reach people is through people” as they can demonstrate what PEARLS is through sharing their experiences about how the program helped them. Motivational interviewing techniques are also useful for engaging participants who are reluctant to join the program.

## Effectiveness

Since the original PEARLS study, PEARLS continues to show positive results in older adults with major depression, with all-age adults, with veterans and vet’s spouses or widows, and with elders with low literacy and with limited English proficiency. PEARLS has been implemented with bicultural, bilingual counselors in Hispanic, Chinese, Vietnamese, Korean, and Filipino communities, and using trained medical interpreters with Somali and Russian-speaking elders. More recent studies demonstrate that the improvement in depressive symptoms extended for 18 months following baseline (14). We often hear stories on our technical assistance calls about how PEARLS benefits a participant’s life, such as helping a client change their blood pressure medication to minimize side effects, submitting paperwork for subsidized housing, or getting a respite care worker to come in 1 day a week. As one 95-year-old participant put it, “PEARLS rocks,” as he now does 50 repetitions on his rocking chair for physical activity. Immigrant elders that participate in PEARLS have overcome loneliness and homesickness, feel more self-sufficient, independent, an overall sense of dignity, and at “peace-of-mind,” and acculturate more quickly into their new community through social contacts and physical fitness. PEARLS participants have also identified how the PEARLS process and worksheet helped them to improve their focus on certain issues and their ability to prioritize and plan, thus, feeling more control over things that had once seemed quite scattered (15).

## Adoption

During the initial PEARLS research study, master’s level social workers and nurses were trained to deliver the intervention. A geriatric psychiatrist provided clinical supervision. In practice, bachelor’s level case managers and social work interns have successfully implemented PEARLS. They may not only require more supervision up front (such as with administering the PHQ-9) but also come to PEARLS with less ingrained therapeutic modalities that may need to be put aside when delivering a structured, participant-driven protocol like PEARLS. A clinical psychologist or other clinician with experience in late-life depression and problem-solving treatment can provide clinical supervision, along with a medical provider who brings expertise on co-occurring chronic conditions and medication use. Community mental health agencies can offer PEARLS as part of their menu of options for persons with mental illness. PEARLS may also be a first step in a person’s depression treatment, using the PEARLS sessions to identify appropriate and accessible longer-term treatment options after the brief intervention ends.

## Implementation

One of the reasons that evidence-based programs are adopted is because research shows them to be effective. Thus, there is a concern that fidelity to the research model be maintained when implementing the program. We developed a PEARLS fidelity instrument to assist in measuring fidelity and found that differences exist for clinical supervision, counselor assessment, client eligibility, and some content and format of PEARLS sessions (16). We do not necessarily view this as a negative since programs need to adapt PEARLS to fit their local implementation environment.

Whether a person is appropriate for PEARLS is one of the most common questions we get on our technical assistance calls. In practice, organizations see complex clients who often do not have any other acceptable options for depression treatment. Expanding eligibility criteria may require adaptations; for example, focusing on increasing physical and social activities rather than problem-solving for participants with mild cognitive impairment or for those for whom a problem-solving approach is not a cultural norm. Some adaptations occur naturally as PEARLS spreads across the country in diverse settings and communities. Strategies for working with low-literacy participants include reading worksheets aloud and having the counselor or caregiver help fill out the worksheet, being mindful of what is written when others will read the worksheets.

## Maintenance

The Program to Encourage Active, Rewarding Lives is now active in 50 agencies across 18 states. Some agencies have only begun implementing the program in the past 6 months while others are over 10 years old. Sustainable funding for PEARLS remains a challenge yet successes such as the California “Millionaire’s Tax” supporting the Mental Health Services Act, prevention and early intervention (PEI) funding in Los Angeles, and a property tax levy and a Medicaid waiver in Washington State hold promise. The PEARLS training program continues to support new and existing program needs including an online component and site-based trainings. Former PEARLS program participants are being engaged to spread the word about PEARLS in their communities.

There are many opportunities for continuing to improve how PEARLS is delivered and spread across the country and beyond. While PEARLS programs continue to spread across the country, this dissemination pattern is more the result of passive diffusion and a by-product of the ongoing training program and PEARLS inclusion in several evidence-based practice registries. We need future research of more active dissemination approaches (such as policy-level interventions) coupled with ongoing dissemination and implementation research for overcoming challenges. An economic evaluation of the program through formal cost effectiveness or return on investment (ROI) analyses might facilitate broader dissemination activities. Future directions for PEARLS also include building capacity through expanding online and regional training options. With continued interest in fidelity assessment, a larger validation study of the PEARLS fidelity instrument is needed to establish the validity of the items as well as the innovative methodological approach of having a self-reported fidelity assessment. Exploring the relationship of fidelity to client outcomes could then follow and allow for refinement of the instrument and better elucidation of the key components of PEARLS to guide program adaptation to best fit local implementation needs. Addressing these needs will help PEARLS achieve its full potential in improving the lives of older adults.

## Conflict of Interest Statement

The authors declare that the research was conducted in the absence of any commercial or financial relationships that could be construed as a potential conflict of interest.

This paper is included in the Research Topic, “Evidence-Based Programming for Older Adults.” This Research Topic received partial funding from multiple government and private organizations/agencies; however, the views, findings, and conclusions in these articles are those of the authors and do not necessarily represent the official position of these organizations/agencies. All papers published in the Research Topic received peer review from members of the Frontiers in Public Health (Public Health Education and Promotion section) panel of Review Editors. Because this Research Topic represents work closely associated with a nationwide evidence-based movement in the US, many of the authors and/or Review Editors may have worked together previously in some fashion. Review Editors were purposively selected based on their expertise with evaluation and/or evidence-based programming for older adults. Review Editors were independent of named authors on any given article published in this volume.
